# Reviews

**Published:** 1881-03

**Authors:** 


### Reviews.

---

**A Treatise of the Principles and Practice of Medicine; designed for the Use of Practitioners and Students of Medicine** By AUSTIN FLINT, M. D. Fifth Edition, revised and largely re-written. Philadelphia: Henry C. Lea's Son & Co. 1881.

There is, probably, no other medical text book so well known in this section of the country as Flint on Practice. This being the case, a review is necessarily a useless task. But the present edition possesses many improvements upon the former and to these we briefly call attention. In the words of the author, "among these changes are the introduction of a new section, devoted to the diseases of the hæmatopoietic system, the classification of the diseases of the nervous system upon an anatomical, in place of a symptomatic basis, a fuller consideration of various diseases, and the addition of several which were not considered in the previous editions. In the descriptions of individual diseases larger space is accorded to the morbid anatomy, including histological appearances, and to the pathological character. In short it may be claimed for the present edition that the eliminations, substitutions and additions render it essentially a new work."

As a practical work for the use of practitioners we believe that this treatise has no superior. We feel an active interest in once more calling public attention to its real value.

**A Practical Treatise on Nasal Catarrh.** By BEVERLY ROBINSON, A. M., M. D. (Paris), Lecturer upon Clinical Medicine at the Bellevue Hospital Medical College, N. Y. New York: Wm. Wood & Co., 27 Great Jones street. 1880.

Of late this department of medicine has been enriched by important additions to its literature. Such works as Cohen in this country, and Mackenzie in England, have given the profession most exhaustive treatises. In the present work the author aims to treat simply nasal catarrh, and allied diseases. We are especially interested in the chapter on Follicular disease of the naso-pharyngeal space, to which he devotes considerable space. The author claims that post-nasal catarrh is the same as follicular disease of the pharynx, only differing in location. The persistency of these diseases, especially when dependent upon a phthisical diathesis, are well known, and our author gives clear and concise views upon these subjects, which will be of valuable service to the profession. Herein lies the great value of his work as a contribution to the literature of these diseases. We commend the result of his labors, believing it the most valuable work of its kind lately placed before the profession.

---

**Management of Children.** By ANNIE M. HALE. Philadelphia: Presley Blakiston, 1012 Walnut street. 1880.

This is a neat little book of about one hundred pages, intended for the use of mothers. Without being able to give a reason, we were at first prejudiced against it, but more careful examination has resulted in an entire reversal of opinion. We consider the work as one worthy of strong recommendation. The subjects which are touched upon are those regarding which there exists a great amount of popular error. Every physician is often strongly and disagreeably impressed with the want of general knowledge upon the subjects of air, exercise, diet, indigestion, &c., &c. These and many other topics are treated by the author in a practical and common-sense manner. We shall use our influence in the introduction of this work to families under our care, and we urge the profession generally to follow our example.

